# Histone lactylation bridges metabolic reprogramming and epigenetic rewiring in driving carcinogenesis: Oncometabolite fuels oncogenic transcription

**DOI:** 10.1002/ctm2.1614

**Published:** 2024-03-08

**Authors:** Yu Zhang, Hang Song, Meili Li, Peirong Lu

**Affiliations:** ^1^ Department of Clinical Medicine Xuzhou Medical University Xuzhou Jiangsu China; ^2^ Department of Ophthalmology Peking Union Medical College Hospital Beijing China; ^3^ Department of Ophthalmology Eye Disease Prevention and Treatment Institute of Xuzhou, The Affiliated Xuzhou Municipal Hospital of Xuzhou Medical University Xuzhou First People's Hospital Xuzhou Jiangsu China; ^4^ Department of Ophthalmology The First Affiliated Hospital of Soochow University Suzhou China

**Keywords:** cancer, histone lactylation, metabolic reprogramming

## Abstract

Heightened lactate production in cancer cells has been linked to various cellular mechanisms such as angiogenesis, hypoxia, macrophage polarisation and T‐cell dysfunction. The lactate‐induced lactylation of histone lysine residues is noteworthy, as it functions as an epigenetic modification that directly augments gene transcription from chromatin. This epigenetic modification originating from lactate effectively fosters a reliance on transcription, thereby expediting tumour progression and development. Herein, this review explores the correlation between histone lactylation and cancer characteristics, revealing histone lactylation as an innovative epigenetic process that enhances the vulnerability of cells to malignancy. Moreover, it is imperative to acknowledge the paramount importance of acknowledging innovative therapeutic methodologies for proficiently managing cancer by precisely targeting lactate signalling. This comprehensive review illuminates a crucial yet inadequately investigated aspect of histone lactylation, providing valuable insights into its clinical ramifications and prospective therapeutic interventions centred on lactylation.

## Graphical abstract

1. The lactate‐induced lactylation of histone lysine residues bridges the metabolic reprogramming and epigenetic rewiring.
2. Histone lactylation fuels oncogene overexpression, expediting tumour progression and development.
3. Targeting lactate signalling exhibits with therapeutic efficacy in diversified cancers.

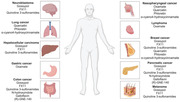

## 1 INTRODUCTION

In the past, lactate was primarily regarded as an outcome of energy metabolism; nevertheless, its distinctive biological significance has been progressively comprehended subsequent to the identification of the Warburg effect.^1^ As a result of increased glycolytic levels, lactate plays various roles in promoting cancer, such as being used as a source of energy metabolism, participating in signal transduction pathways, regulating the tumour microenvironment (TME) and immune cells, and regulating specific enzyme modifications.^2^, ^3^ Consequently, lactate is involved in a wide range of biological aspects related to tumour regulation, including energy transport and growth.^4^, ^5^, ^6^

Multiple studies conducted over several decades have consistently demonstrated that changes in patterns of gene expression are primarily attributed to the process of chromatin remodelling and posttranslational modifications (PTMs), commonly referred to as epigenetic alterations.^7^, ^8^, ^9^ The alterations, which have the ability to be passed down and undone, have a significant impact on the specific control of cellular characteristics.^10^, ^11^ As a result, a variety of chromatin modifications, including methylation, acetylation, phosphorylation, ubiquitination, glycosylation and glutarylation, collectively contribute to distinct configurations that impact the efficacy of transcriptional machinery.^12^, ^13^, ^14^ Significantly, the disturbance of epigenetic alterations and metabolic pathways is frequently linked to various illnesses, and the interaction between metabolism and chromatin presents numerous prospects for therapeutic interventions.^15^, ^16^

Recognising the interaction between metabolic remodelling and dynamic histone tuning is of utmost importance as it is currently in its early stages. The purpose of this review is to combine current information on the influence of lactate and histone modification on tumour and related gene regulation. Furthermore, we have clarified the scientific significance of future studies on histone alteration and recognised the obstacles that must be tackled, thus opening up new possibilities in the realm of cancer management through epigenetic/metabolic therapies.

**FIGURE 1:**
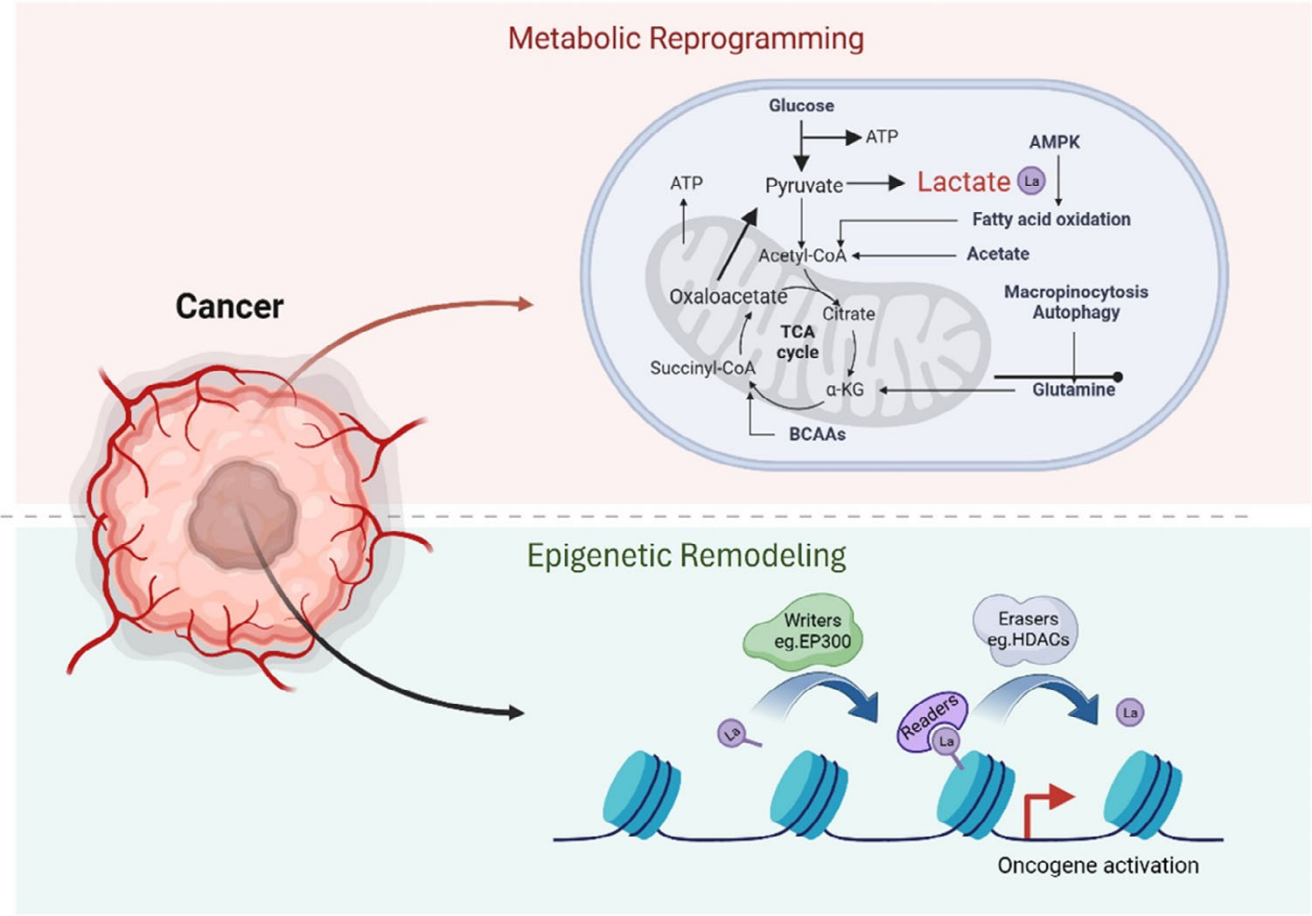
The correlation between histone lactylation and the interconnection of epigenetic remodelling and metabolic reprogramming during the progression of cancerous development. Metabolic disorders are visually represented in red, while epigenetic rewiring is depicted in green.

In this review, we introduce a newly discovered epigenetic modification called histone lactylation modification, which is a dynamic and reversible epigenetic modification dependent on lactate.^17^, ^18^, ^19^ Examining histone lactylation provides a thorough analysis of the latest advancements in researching this alteration in cancer, particularly emphasising its effects on reshaping tumour characteristics, regulating gene activity, promoting glycolysis in cancer stem cells and impacting the tumour's immune microenvironment (Figure 1).^20^, ^21^, ^22^

### 1.1 Warburg effect

For the aerobic respiration, pyruvate derived from glucose is transported into the mitochondria and undergoes conversion to acetyl‐CoA. Afterward, it proceeds into the tricarboxylic acid (TCA) cycle, which aids in oxidative phosphorylation (OXPHOS).^23^, ^24^, ^25^, ^26^, ^27^, ^28^, ^29^, ^30^ Through this process, a grand sum of 36 adenosine triphosphate molecules are generated for each glucose molecule.^31^, ^32^, ^33^ Instead of entering the TCA cycle, the pyruvate molecules are converted to lactate through the catalytic activity of cytosolic lactate dehydrogenases (LDHs).^34^

In 1956, Otto Heinrich Warburg made the seminal observation that substantial lactate production occurs from glucose, particularly in tumour cells, a phenomenon now referred to as the Warburg effect.^19^, ^35^ The Warburg effect is distinguished by elevated glycolysis and diminished OXPHOS, conferring specific advantages to cancer cells.^36^, ^37^, ^38^, ^39^ In addition, glycolysis generates intermediate compounds that aid in the synthesis of nucleotides, proteins and fats, regardless of changes in oxygen concentrations.^40^ Furthermore, the sustenance of glycolysis relies on maintaining the ratio of nicotinamide adenine dinucleotide (oxidative) (NAD^+^) to nicotinamide adenine dinucleotide (reductive) (NADH), which is achieved by converting pyruvate to lactate and then regenerating NAD^+^.^41^, ^42^, ^43^ In conclusion, improved glycolysis promotes increased lactate generation, resulting in the acidification of the cellular surroundings.

To support their growth, survival, proliferation and long‐term maintenance, cancer cells undergo this metabolic reprogramming.^44^, ^45^ An important feature of this altered metabolic condition is the increased absorption of glucose and subsequent transformation of glucose into lactate, leading to the acidic environment of the TME.^46^, ^47^, ^48^, ^49^ Furthermore, hypoxia plays a crucial role in promoting glycolysis and inhibiting OXPHOS, which is frequently observed in cancerous tissues. These aforementioned observations emphasise the role of lactate signalling in the development of tumours.^31^, ^50^

### 1.2 Lactate production and elimination

During the process of Warburg effect, a substantial quantity of lactate accumulates as a consequence of this metabolic reprogramming phenomenon.^51^, ^52^, ^53^ Importantly, the buildup of lactate in the human organism presents a higher danger in contrast to the buildup of alternative molecular energy sources, since elevated levels of lactate in the bloodstream can lead to lactic acidosis.^42^, ^54^ The enzymatic activity of pyruvate dehydrogenase (PDH) enables the conversion of lactate to pyruvate, resulting in the elimination process.^55^, ^56^, ^57^, ^58^ Moreover, the buildup of lactate has the potential to trigger gluconeogenesis in cells of the liver and skeletal muscle. During energy expenditure, lactate is converted into glucose and subsequently released into the bloodstream to facilitate further glucose consumption.^59^, ^60^, ^61^

Furthermore, alongside glycolysis, the process of glutamine catabolism serves as an additional approach for cancer cells to produce lactate.^61^ The c‐Myc protein controls this metabolic pathway, enabling the movement of glutamine undergoes enzymatic conversion into glutamate through the action of the enzyme glutaminase.^62^, ^63^, ^64^ Afterwards, the transformation of glutamate into α‐ketoglutarate takes place via the enzymatic activity of glutamate dehydrogenase or a cluster of transaminases.^65^, ^66^ During this process, carbon obtained from glutamine is converted into oxaloacetate, which is then changed into malate and leaves the mitochondria to be further converted into NADPH and pyruvate.^67^, ^68^

### 1.3 Lactate sensing, shuttling and signalling

Importantly, the transportation and detection of lactate rely on the utilisation of monocarboxylate transporter (MCT) and sodium MCT families, along with G protein‐coupled receptors (GPRs) called GPR81 and GPR132.^69^ In cancer cells, glutamine serves as a vital carbon skeleton for the production of lactate and acts as an additional pathway for generating lactate. Out of the 14 recognised MCTs, MCT1–4 are present in different body tissues and play a role in the catalytic coupling of protons and the two‐way movement of monocarboxylic acid.^70^

Under physiological conditions, the combined functioning of MCT1–4 facilitates the transfer of lactate between cells that undergo glycolysis and those that undergo oxidation, playing a crucial role in maintaining lactate balance in various tissues.^71^ The MCT1 with strong affinity helps maintain balance of lactate by facilitating the movement of lactate across the cell membrane in response to the transmembrane lactate gradient.^72^ Tumour cells and other cells with elevated levels of lactate within rely on MCT4, which has a low affinity for transporting lactate. The transportation process starts by attaching unbound protons to MCT, then proceeds with the attachment of lactate, which experiences a structural alteration inside the carrier and is subsequently released on the opposite side of the membrane.^30^, ^73^ Lactate is released prior to the release of protons. Of particular significance, increased levels of MCT1, MCT2 and MCT4 are strongly linked to the development of cancer, which is correlated with an adverse prognosis.^74^, ^75^, ^76^, ^77^ The transport of lactate, enabled by MCTs, creates internal links, and has a vital function in the collaborative metabolism of glycolytic cancer cells and oxidative cancer cells, ultimately aiding in the onset and advancement of tumours.^78^

Classical metabolites have the ability to initiate direct signalling via GPRs.^79^ Upon the binding of an agonist, the G protein‐coupled receptor (GPCR) alpha subunit undergoes a GDP–GTP exchange. Secondary messengers such as cyclic adenosine monophosphate (cAMP) and Ca^2+^ play a role in the following signalling events, and their variation depends on the specific alpha subunit type.^80^, ^81^ Notably, lactate is capable of signalling through GPR81 and GPR132, both of which are sensitive to protons.^82^

**FIGURE 2:**
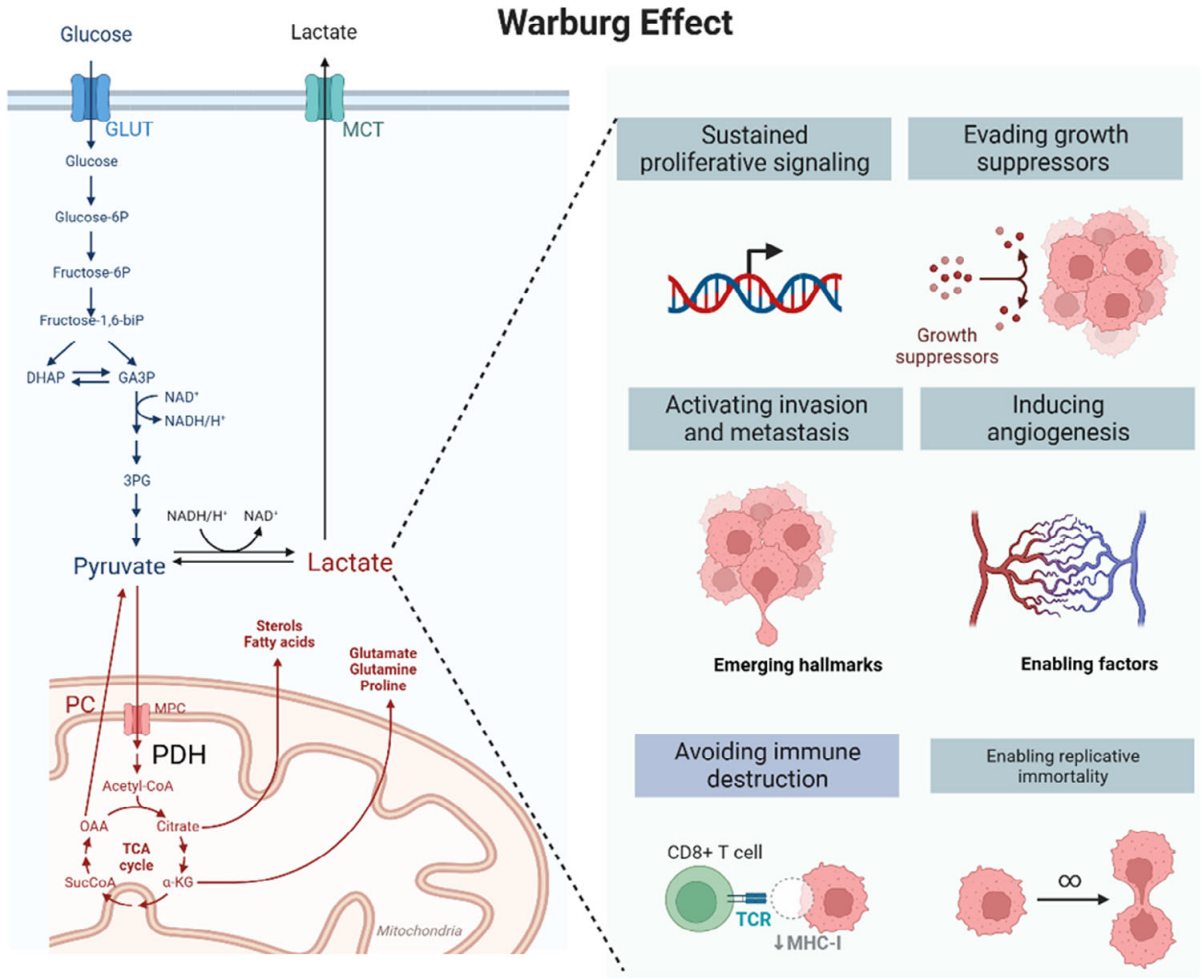
The illustration of the Warburg effect elicits an elevation in lactate levels, which is associated with multiple cancer‐related traits inclding enhanced signalling for cell proliferation, evasion of growth inhibitory factors, activation of invasion and metastasis, heightened capacity for angiogenesis, evasion of immune system destruction and facilitation of replicative immortality.

GPR81 is detected in different tumours obtained from patients and cancer cell lines. In vivo studies have demonstrated a correlation between levels of GPR81 and tumour growth and metastasis in pancreatic cancer, with a significant decrease observed upon silencing of GPR81.^83^ Crucially, lactate has been shown to actively promote the formation of new blood vessels by activating the phosphoinositide 3‐kinase/Akt (PI3K/Akt)–cAMP–CREB (cAMP response element‐binding protein) pathway, resulting in the production of pro‐angiogenic amphiregulin in breast cancer.^84^, ^85^ Moreover, lactate has been found to impede the efficiency of anti‐cancer immune response by communicating through GPR81 in dendritic cells that invade tumours, thereby compromising their capacity to effectively showcase antigens to T cells by reducing the expression of major histocompatibility complex class II (MHC‐II) molecules.^86^ Furthermore, GPR132 is detected in different body tissues, including the respiratory system, digestive system and immune cells, specifically macrophages.^87^ It is worth mentioning that the stimulation of GPR132 through lactate signalling in macrophages associated with tumours has been discovered to enhance a pro‐tumoural M2 phenotype, which is identified as alternatively activated and anti‐inflammatory, in models of breast cancer and Lewis lung carcinoma.^88^, ^89^, ^90^ The expression of GPR132 exhibits a positive correlation with the existence of M2 macrophages and the incidence of metastasis.^91^ (Figure 2)

## 2 THE ROLE OF HISTONE LACTYLATION

**FIGURE 3:**
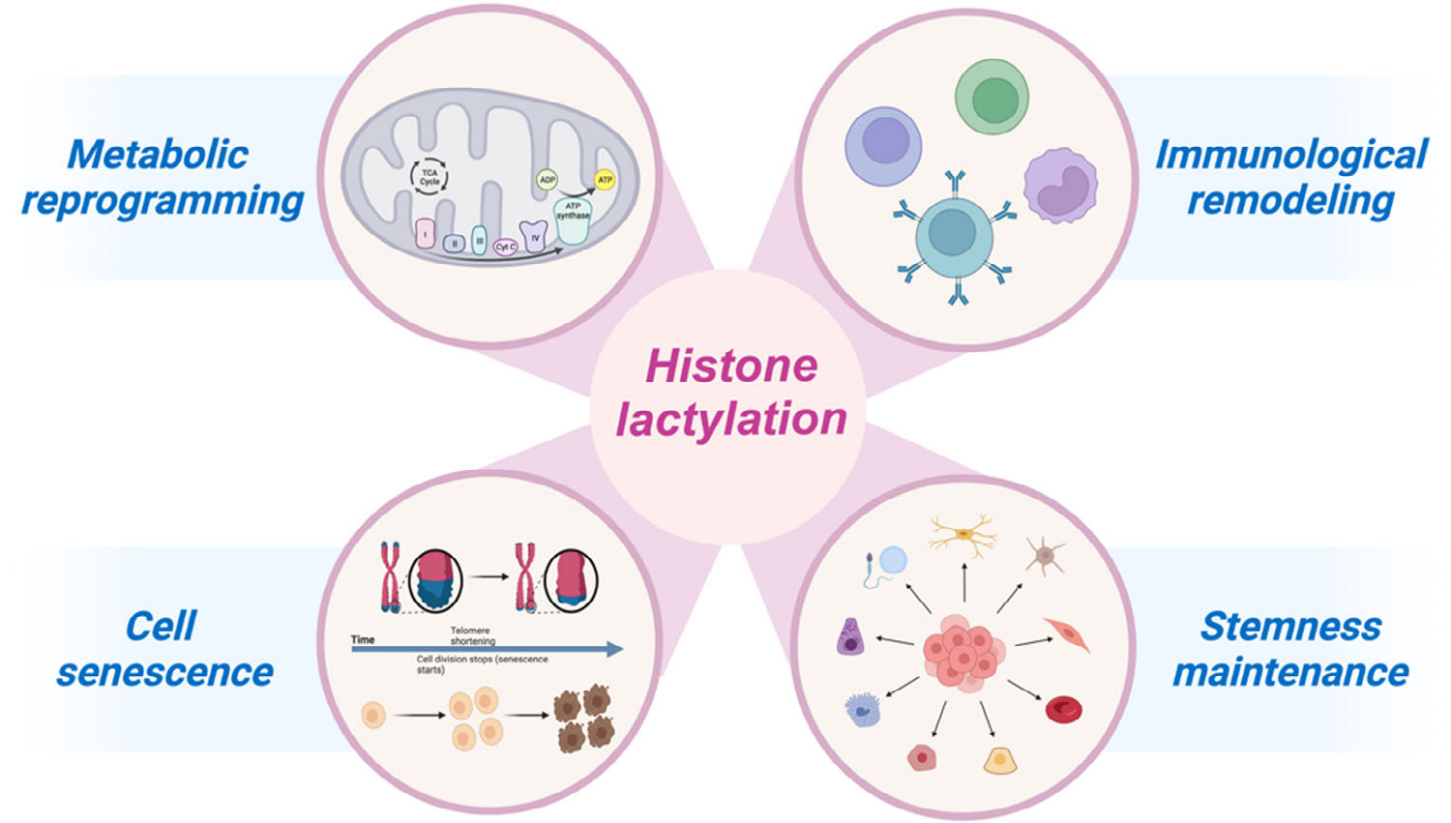
Oncogenic functions of histone lactylation in metabolic reprogramming, immunological remodelling, cell senescence and stemness maintenance.

While the significance of lactate as a catalyst for oncogenic signalling has been established, its role in transcriptional regulation remains uncertain. Notably, substantial evidence supports the crucial function of lactate in aiding the alteration of histone lysine residues. This alteration, such as other modifications of histones, plays a role in the regulation of transcriptional activation.^19^ Thus far, the importance of histone lactylation in facilitating metabolic rewiring, altering the immunological environment, controlling cell destiny and impacting cancer stemness and senescence has been discovered (Figure 3 and Table 1).^92^, ^93^, ^94^

**TABLE 1:** The role of histone lactylation.

Downstream targets	Cells	Function	Reference
Bmp5, Trpc5, Kit	PASMC	Promotes PASMC proliferation and vascular remodelling in hypoxic pulmonary hypertension	^96^
YTHDF2	OCM1, CRMM1	Drives ocular melanoma development	^94^
–	*Saccharomyces cerevisiae*	Attenuates dextran sulphate sodium‐induced colitis via suppressing macrophage pyroptosis and modulating the intestinal microbiota	^102^
METTL3	Tumour infiltrating myeloid cells	Strengthens immunosuppressive functions of myeloid cells	^130^
PD‐L1	HL‐60, NB4	Suppresses T‐cell activation in acute myeloid leukaemia	^21^
Lrg1, Vegfa, IL10	Monocyte	Promotes early remote activation of the reparative transcriptional response in monocytes post‐myocardial infarction	^105^
Rela, NFκB1	Microglia	Upregulates senescence‐associated secretory phenotype and accelerates the pathogenesis of Alzheimer's disease	^100^
Oct4, Sall4, Mycn	iPSC	Facilitates cellular reprogramming	^114^

Abbreviations: CRMM1, Conjunctival recurrent malignant melanoma 1; IL10, interleukin 10; METTL3, Methyltransferase Like 3; NFκB1, Nuclear Factor of Kappa Light Polypeptide Gene Enhancer in B Cells 1; OCM1, Ocular chroidal melanoma 1; PASMC, pulmonary artery smooth muscle cell; PD‐L1, programmed death‐ligand 1; YTHDF2, YTH N6‐methyladenosine RNA‐binding protein 2.

### 2.1 Histone lactylation and metabolic reprogramming

The regulation of lactylation by lactate has recently been established as a novel factor influencing the epigenetic landscape.^18^, ^26^ This discovery not only paves the way for comprehensive investigations into lactate metabolism but also provides crucial points of interest for future studies on functionality and mechanisms. Enhanced glycolysis and the accumulation of lactate are commonly observed in various types of cancer.^47^, ^73^, ^85^ This metabolic shift leads to the intracellular production of lactate, which has been found to drive a recently discovered PTM known as lysine lactylation on core histones. A recent investigation performed worldwide lactylome profiling on a group of hepatitis B virus‐related hepatocellular carcinoma (HCC) and nearby liver tissues that were collected in advance.^95^

By conducting an integrative analysis of lactylome and proteome, a grand total of 9275 Kla sites were discovered, out of which 9256 sites were found on proteins other than histones. The results suggest that Kla is a widespread alteration that goes beyond histone proteins and the regulation of transcription. Notably, Kla demonstrates a predilection for influencing enzymes involved in diverse metabolic pathways, including the TCA cycle, as well as carbohydrate, amino acid, fatty acid and nucleotide metabolism.^95^ Moreover, in the framework of the pulmonary hypertension (PH) model, Chen et al. observed an augmentation of histone lactylation on hypoxia inducible factor 1 alpha (HIF‐1α) targets, namely Bmp5, Trpc5 and Kit, which subsequently stimulates the proliferation of pulmonary artery smooth muscle cells (PASMCs). Furthermore, the use of a LDH inhibitor as a pharmacological treatment led to a decrease in histone lactylation, which ultimately enhanced the proliferation of PASMC and vascular remodelling in hypoxic PH rats.^96^

Furthermore, recent research indicates that metabolic adaptation is a crucial characteristic and requirement for the transition of macrophage phenotype.^89^, ^97^ Pyruvate kinase M2 (PKM2) is a fundamental molecular factor in the metabolic adaptations of pro‐inflammatory macrophages.^98^, ^99^ The regulation of PKM2 is primarily influenced by PTMs. Specifically, lactylation of PKM2 hinders its transformation from a tetramer to a dimer, thereby enhancing its pyruvate kinase activity and decreasing its nuclear distribution.^100^ In summary, these findings emphasise the significance of lactylation as a key initiator of metabolic adjustments in diversified pathological events.

In addition, the involvement of histone lactylation has been suggested in the control of diverse forms of chemical alterations. For instance, Yu et al. noticed a connection between increased amounts of histone Kla and an adverse outlook in individuals with ocular melanoma. The strong positive correlation between ocular melanoma and intracellular histone Kla was further confirmed through the subsequent inhibition of histone Kla in ocular melanoma cells. Melanoma development is caused by the increase in YTHDF2 expression, which is achieved by enhancing histone Kla at the promoter region. YTHDF2, an m6A reader, plays a crucial part in enhancing the development of ocular melanoma.^94^ While certain observations endorse the concept that histone Kla is crucial for the proliferation of tumours, alternative studies argue that histone Kla lacks the ability to convert healthy cells into cancerous ones. This suggests that histone Kla plays a role in facilitating the growth of developing tumours rather than initiating tumourigenesis initially.^15^, ^20^

### 2.2 Histone lactylation and immunological remodelling

Lactic acid, a substance produced during metabolism by both the host and intestinal microbiota, has been recognised as a crucial signalling molecule in the immune system.^69^, ^101^ Notably, Sun et al. successfully engineered *Saccharomyces cerevisiae* to produce lactic acid from glucose instead of ethanol, resulting in a high production yield. The application of this genetically modified strain of *S. cerevisiae* has been shown to effectively alleviate dextran sulfate sodium (DSS)‐induced colitis in mice through the inhibition of macrophage pyroptosis and modulation of the intestinal microbiota. This approach holds great potential as a safe and promising therapeutic strategy for the treatment of ulcerative colitis. Howbeit, additional research is needed to determine whether this engineered *S. cerevisiae* strain is also involved in tumourigenesis, particularly in the context of autoimmune diseases.^102^

The rapid advancements in genomics and immunology have led to the emergence of immunotherapy as a groundbreaking therapeutic approach for tumour treatment. Immune checkpoint inhibitors, in particular, have demonstrated remarkable efficacy in various tumour types.^19^, ^40^ Histone lactylation was initially discovered in the macrophages present in the TME, which aligns with the observed high glucose uptake in this specific subpopulation.^5^ Furthermore, the increased histone lactylation originating from tumours contributes to the polarisation of M2 tissue‐associated macrophages, thereby promoting immunological evasion during the progression of cancer.^100^, ^103^, ^104^

Recently, histone lactylation is also involved in the response towards immunotherapy. For instance, the promotion of increased lactate accumulation facilitated the nuclear translocation of E3BP, a component of the PDH complex. It is worth noting that E3BP demonstrated the capacity to interact with lactylated histone, leading to significant histone lactylation and ultimately resulting in the upregulation of programmed death‐ligand 1 (PD‐L1) transcription. This finding emphasises the significance of nuclear translocation of metabolic enzymes in connecting histone lactylation and PD‐L1‐mediated T‐cell exhaustion.^21^ Furthermore, the timely activation of reparative signals is necessary for the resolution of inflammation and the initiation of cardiac repair following myocardial infarction. The lactylation of histones facilitates the early activation of the reparative transcriptional response in monocytes, which is crucial for maintaining immune balance and initiating timely cardiac repair after myocardial infarction.^105^

In addition to its essential role in histone lactylation, non‐histone lactylation has been established as a significant mediator in immunological remodelling. A noteworthy illustration of this phenomenon is observed in hepatocyte HSPA12A, which functions as a novel regulator to protect livers against ischemia–reperfusion (I/R) injury. The inhibition of glycolysis‐mediated HMGB1 lactylation and its subsequent secretion from hepatocytes achieves a protective mechanism, which effectively suppresses macrophage chemotaxis and inflammatory activation.^106^

### 2.3 Histone lactylation and cell senescence

Cellular ageing plays a crucial role in the progression of age and is closely linked to various age‐related ailments, such as Alzheimer Disease (AD), retinopathies and tumours.^50^ Comparatively higher concentrations of lactic acid were detected in aged mice and AD mouse models' senescent microglia and hippocampus tissues, when compared to their corresponding counterparts. Moreover, the levels of H3K18 lactylation and Pan‐Kla were notably increased in both senescent microglia and hippocampus tissues of naturally aged mice as well as AD modelling mice. The results suggest that focusing on this pathway may be a viable approach to slow down the ageing process and delay the onset of AD by reducing the effects of the senescence‐related secretory phenotype.^100^, ^107^

Hair follicle stem cells (HFSCs) undergo rapid activation and division during a new hair cycle. The quiescence of HFSCs is regulated by various intrinsic and extrinsic mechanisms as individuals age. HFSCs employ glycolytic metabolism and exhibit higher lactate production compared to other epidermal cells. Moreover, lactate generation plays a crucial role in the activation of HFSCs, as evidenced by the prevention of their activation upon deletion of LDH. While this study primarily focuses on the involvement of lactate in HFSC activation, further investigation is needed to explore the role of lactylation in successive studies.^108^

Furthermore, it is important to note that the microenvironment within cancerous tissues exhibits immunosuppressive and pro‐tumourigenic characteristics, often characterised by elevated levels of senescence‐associated secretory phenotype factors across various cancer types.^109^ In contrast, tissues impacted by chronic inflammatory diseases exhibit a pro‐inflammatory milieu that impedes the resolution mechanisms.^87^ Despite the divergent immunological conditions, the metabolic profiles within the tissue microenvironments of cancer and inflammatory diseases are analogous: both are characterised by hypoxia, heightened lactate levels and other metabolic by‐products, as well as diminished nutrient levels.^110^, ^111^ While several studies have suggested the substantial involvement of ageing in tumourigenesis, the precise role of histone lactylation remains enigmatic and necessitates further investigation.

### 2.4 Histone lactylation and stemness maintenance

The process of somatic cell reprogramming offers valuable insights into the fundamental mechanisms underlying cell fate determination, particularly in relation to the regulation of stemness.^112^, ^113^ In a study conducted by Li et al., it was demonstrated that Glis1 directly interacts with chromatin, resulting in the activation of glycolytic genes while repressing somatic genes, ultimately leading to an upregulation of glycolysis. Furthermore, this increased glycolytic activity promotes higher levels of cellular acetyl‐CoA and lactate, which in turn enhance acetylation and lactylation at gene loci associated with pluripotency. This process facilitates the opening of these loci, thereby facilitating cellular reprogramming.^114^

Moreover, lactate exhibits the potential to augment the stemness of CD8^+^ T cells and fortify the immune response directed towards tumours. Examination employing single cell transcriptomics reveals an elevated ratio of CD8^+^ T cells expressing the stem‐like T‐cell factor 1 (TCF‐1) marker within intra‐tumoural CD3^+^ cells. This characteristic has been corroborated via in vitro lactate treatment of T cells. The effects of lactate are mediated by the inhibition of histone deacetylase activity, resulting in increased acetylation at the H3K27 site of the *Tcf7* super enhancer locus. This, in turn, leads to the upregulation of Tcf7 gene expression. Conversely, the modulation of abnormal lactate levels through the inhibition of LDH also promotes the stemness of CD8^+^ T cells and initiates immune responses against tumours. Collectively, these findings suggest that lactate may play a dual role in regulating the stemness of T cells.^115^

## 3 CANCER THERAPY TARGETING HISTONE LACTYLATION

**FIGURE 4:**
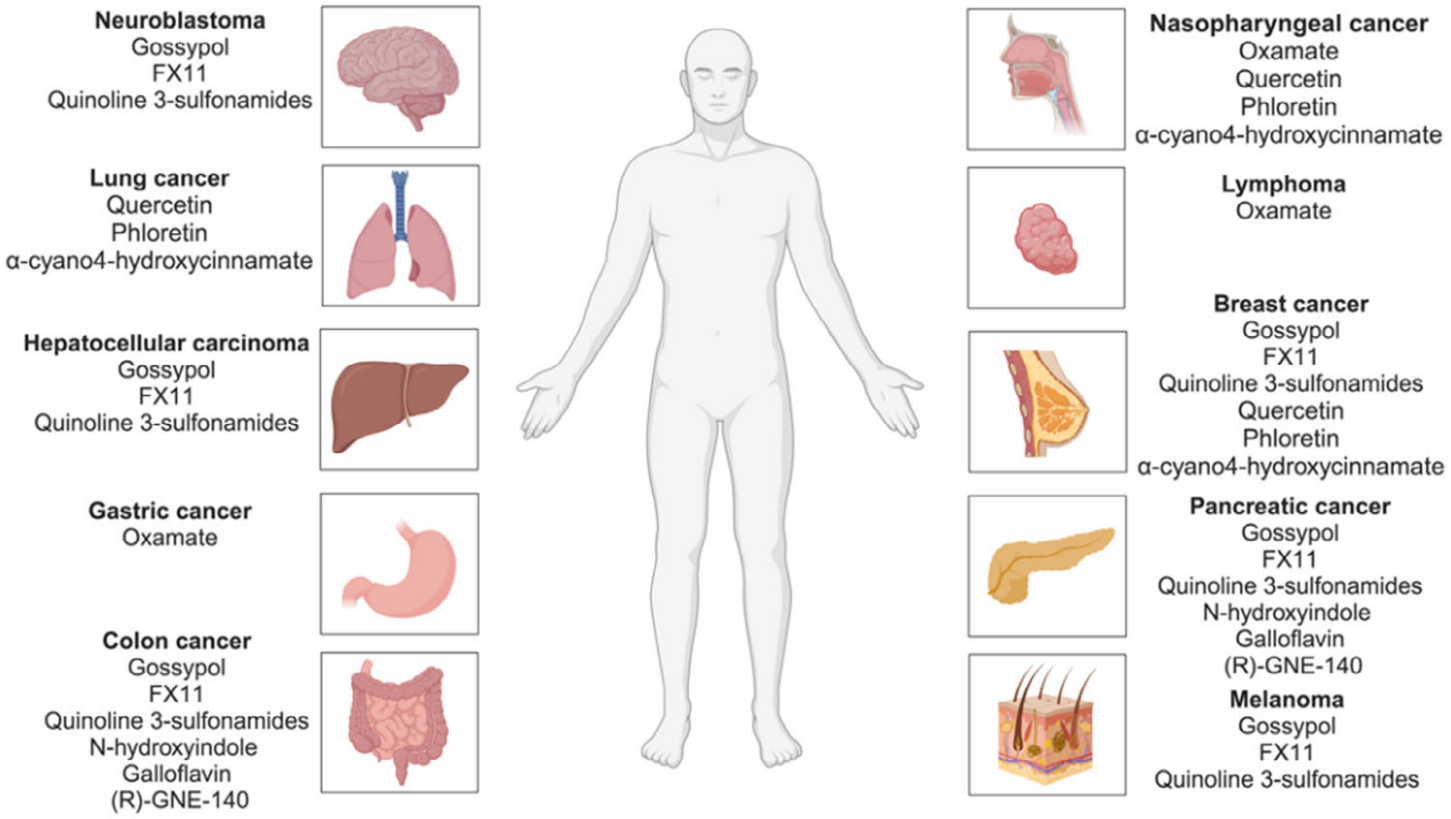
Examples of targeting lactate metabolism in cancer treatment, including neuroblastoma, lung cancer, hepatocellular carcinoma, etc.

During cancerous initiation and progression, lactate has been traditionally regarded as a by‐product of aerobic glycolysis in metabolism.^108^ Nevertheless, an increasing number of studies have indicated that lactate has the ability to control the advancement of cancer through various mechanisms including but not limited to cell cycle control, immune system suppression and energy metabolism. The recent discovery of lactylation has attracted considerable attention and has become a prominent topic of conversation in the realm of cancer studies. While several studies have demonstrated the successful therapeutic efficacy of targeting lactate metabolism in various types of cancer (Figure 4 and Table 2), the specific modulation of histone lactylation remains unresolved.

**TABLE 2:** Histone lactylation targeted drugs in cancer therapy.

Targets	Drugs	Cells and species	Drug dose and exposure time	Reference
LDH	Oxamate	Nasopharyngeal carcinoma cells; human	100 mmol/L; 72 h	^131^
	PS™B	Colon cancer cells; human	50 µmol/L; 24 h	^2^
	N‐hydroxyindole	Pancreatic carcinoma cells; human	100 µmol/L; 48 h	^132^
HK	Lonidamine	Melanoma and breast cancer cells; human	300 µg/mL; 24 h	^126^
	2‐DG	Cervix adenocarcinoma cells; human	10 mmol/L; 72 h	^133^
	WP‐1122	Nude mice implanted with glioblastoma cells; human	2.5 g/kg; 40 days	^134^
GLUT	STF‐31	Glioblastoma cells, oral squamous cell carcinoma cells, cervix adenocarcinoma cells, head neck cancer cells, colon carcinoma cells and osteosarcoma cells; human	100 µmol/L; 24 h	^135^
	WZB117	Glioblastoma cells, oral squamous cell carcinoma cells, cervix adenocarcinoma cells, head neck cancer cells, colon carcinoma cells and osteosarcoma cells; human	100 µmol/L; 24 h	^135^
	BAY‐876	Triple‐negative breast cancer cells; human	1 µmol/L; 5 days	^136^
PFKFB3	3‐PO	Jurkat cells (T leukaemia cells); human	10 µmol/L; 36 h	^137^
	PFK‐158	Small cell lung cancer cells; human	20 µmol/L; 24 h	^138^
	AZ‐67	Triple‐negative breast cancer cells; human	15 nmol/L; 48 h	^139^
PKM	Compound 3K	Ovarian cancer cells; human	15 µmol/L; 48 h	^140^
	Shikonin	Ovarian cancer cells; human	1.5 µmol/L; 48 h	^141^
	Alkannin	Ovarian cancer cells; human	20 µmol/L; 12 h	^142^
SGLT	Phlorizin	Esophageal squamous cell carcinoma cells; human	1.6 mmol/L; 72 h	^143^
	Canagliflozin	Hepatocellular carcinoma cells; human	15 µmol/L; 48 h	^144^
	Dapagliflozin	Colon cancer cells; human	.5 mmol/L; 35 min	^145^
MCT	Simvastatin	Triple‐negative breast cancer cells; human	20 µg/mL; 48 h	^146^
	Quercetin	Colorectal cancer cells; human	200 mmol/L; 24 h	^147^
	DIDS	Prostate cancer cells; human	1 mmol/L; 48 h	^148^

Abbreviations: HK, Hexokinase; LDH, lactate dehydrogenase; MCT, monocarboxylate transporter; PFKFB3, 6‐Phosphofructo‐2‐Kinase/Fructose‐2,6‐Bisphosphatase 3; PKM, pyruvate kinase M; SGLT, Sodium‐Glucose Cotransporter 1.

### 3.1 Targeting LDHs for cancer treatment

The LDH enzyme complex, composed of LDHA and LDHB subunits, has five different isoforms that are determined by the combination of LDHA and LDHB subunits.^47^ The reduction of pyruvate to lactate is catalysed by LDHA, which occurs simultaneously with the regeneration of NAD+. On the other hand, LDHB is responsible for mediating the reverse reaction. Significantly, LDHA is highly expressed in various tumour tissues and is associated with adverse prognostic outcomes in individuals with cancer.^98^

Preclinical trials have evaluated various LDHA inhibitors for their effectiveness. Oxamate, a compound similar to pyruvate and a competitive inhibitor of LDH, hinders the transformation of pyruvate into lactate, thereby affecting cellular glycolysis.^116^ Studies have recorded that the application of oxamate to lymphoma cells hinders the advancement of acute lymphoblastic leukaemia cells Jurkat and DU528.^117^ According to records, oxamate has been observed to hinder the growth of cells in nasopharyngeal and gastric cancer.^118^ Gossypol, FX11 and quinoline 3‐sulphonamides are part of the group of LDHA inhibitors that compete with nicotinamide adenine dinucleotide. Several research studies have extensively recorded the effectiveness of these in inhibiting the proliferation of different types of cancer cells, such as those found in the colon, neuroblastoma, HCC, primary pancreatic cancer, melanoma and breast cancer.^118^ Furthermore, N‐hydroxyindole‐based inhibitor of lactated dehydrogenase (NHI), galloflavin and [(R)‐3‐((2‐chlorophenyl)thio)‐4‐hydroxy‐6‐(4‐morpholinophenyl)‐6‐(thiophen‐3‐yl)‐5,6‐dihydropyridin‐2(1H)‐one] ((R)‐GNE‐140), functioning as LDHA inhibitors that compete with NADH, have exhibited the capability to impede the growth of cancerous cells, particularly those found in pancreatic and colorectal cancer.

Several specific compounds, such as 1‐(phenylseleno)‐4‐(trifluoromethyl) benzene (PSTMB), phthalimide and dibenzofuran derivatives, have shown the ability to function as LDHA inhibitors, effectively impeding the development and multiplication of cancer cells.^119^ Particularly noteworthy are the novel LDH inhibitors, phthalimide and dibenzofuran derivatives, which display selective inhibition towards the LDHA isoenzyme.^120^ Furthermore, recent studies have emphasised the inhibitory properties of small interfering RNAs (siRNAs) targeting LDHA. The investigation into the utilisation of siRNAs to specifically target LDHA with the intention of impeding the progression of cancer by inducing oxidative stress and cell death has demonstrated their potential as a therapeutic strategy for LDHA‐dependent malignancies.^121^ Furthermore, the oncogenic driver Ewing's sarcoma breakpoint region 1‐friend leukemia virus integration 1 (EWS‐FLI1) in Ewing sarcoma has been observed to impede the proliferation of Ewing sarcoma cells by targeting the transcription of LDHA.^122^ Consequently, these discoveries provide a solid basis for the advancement of LDHA inhibitors. Nevertheless, the development of more potent and selective compounds remains a significant obstacle that must be overcome to facilitate their clinical application.

### 3.2 Targeting MCTs for cancer treatment

MCTs are members of the solute carrier (SLC) transporter family, which encompasses 52 distinct membrane transporter families. Within this group, MCT1 and MCT4 play a pivotal role in the uptake and release of lactate, as well as the transportation of other monocarboxylates including pyruvate, β‐hydroxybutyrate and acetate.^69^, ^123^ The efficacy of lactate transport is contingent upon the intracellular and extracellular concentrations of lactate, pH levels and the concentrations of other MCT substrates. Multiple types of human cancer, including glioma, breast, colorectal, gastric, cervical and neuroblastoma, exhibit heightened expression of MCT1 and MCT4, which has been linked to unfavourable prognostic outcomes.^124^ Inhibition of MCT4 prompts the buildup of intracellular lactate and consequent demise of hypoxic cancer cells. Experimental suppression of MCT4 has demonstrated its essential role in the migratory and invasive capabilities of lung cancer cells.^125^ Consequently, directing therapeutic efforts towards MCTs holds promise as a viable strategy for cancer treatment.

Numerous MCT blockers have exhibited effectiveness in preclinical experiments. Various malignancies, such as cervical cancer, breast cancer, colorectal cancer, prostate cancer, pharyngeal squamous cell carcinoma and other types of cancer, have been effectively treated using classical inhibitors of MCT1/MCT4, such as quercetin, phloretin and α‐cyano4‐hydroxycinnamate.^126^ Additionally, lonidamine, DIDS, simvastatin and other MCT inhibitors have exhibited therapeutic potential for a range of cancer types.^126^, ^127^ Additionally, the chaperone protein CD147, which is common to both MCT1 and MCT4, has been observed to promote the migration, invasion and metastasis of cancer cells. Therefore, the targeting of CD147 offers a promising and innovative strategy for suppressing the function of both transporters.^128^ Notably, p‐chloromercuric besylate, an organomercury compound, and AC‐73, which selectively hinders CD147 dimerisation, are among the compounds that disrupt MCT binding to CD147. Nevertheless, the clinical utilisation of CD147 is constrained by its involvement as a co‐chaperone for other membrane proteins, thereby prompting concerns regarding safety.^129^

## 4 CONCLUSIONS AND FUTURE DIRECTIONS

Lactate serves as both a terminus of glycolysis and a universal metabolic fuel for energy, as evidenced by the emerging concept of the ‘lactate shuttle’ facilitating its movement between cells and transmission of signals.^89^, ^95^, ^108^ The prominence of glycolytic‐dependent metabolism in tumours and rapidly proliferating cells has positioned lactate as a crucial participant in the reprogramming of energy metabolism, allowing cells to efficiently acquire ample energy within a limited timeframe.^60^, ^126^ In addition, lactate has the potential to create advantageous circumstances for the development of tumours through its influence on the acidic microenvironment of the tumour, the recruitment of immune cells, and other mechanisms.

In addition to its fundamental role in accelerating tumourigenesis, histone lactylation also serves as a significant diagnostic marker in various cancers, such as ocular melanoma,^94^ colorectal cancer^4^ and HCC.^95^ Notably, elevated levels of global lactylation (pan‐lactylation) are specifically upregulated in tumour tissues, indicating a higher likelihood of earlier recurrence and increased aggressiveness in ocular melanomas.^94^ Furthermore, there is a significant correlation between elevated levels of pan‐lactylation and H3K18la and decreased overall survival rates in individuals diagnosed with colorectal cancer.^4^ Additionally, it has been observed that HCC patients also exhibit heightened lactylation levels, resulting in the subsequent upregulation of adenylate kinase 2 lactylation. This molecular alteration further promotes the proliferation and metastasis of HCC.^95^ Cumulatively, these findings substantiate the notion that aberrant histone lactylation is significantly elevated across diverse tumour classifications, thus exerting a pivotal influence on the malignant progression of cancer. Nonetheless, the absence of a comprehensive evaluation regarding the disparity in histone lactylation levels between metastatic sites and primary tumours necessitates further investigation, thereby warranting future exploratory endeavours.

Furthermore, the newly discovered occurrence of lactate‐induced lactylation enhances our comprehension of the pro‐tumourigenesis consequences of lactate generation, circulation and utilisation.^66^, ^128^ Analogous to other epigenetic alterations, lactylation possesses the capacity to alter histone proteins, thereby influencing the spatial organisation of chromatin, affecting DNA accessibility and governing the expression of related genes. Moreover, the degree of lactylation is intricately associated with the localised concentration of lactate, establishing a correlation between epigenetics and metabolic reprogramming.^18^, ^19^, ^109^

Significantly, this field encounters numerous challenges. Primarily, histone lactylation exhibits shared ‘writers’ and ‘erasers’ with other types of histone modifications, rendering the specific modulation of histone lactylation notably difficult. For example, most studies have silenced EP300 to reduce histone lactylation level, which simultaneously reduced the global acetylation levels. Therefore, it is important to find a novel method to specifically modulate global lactate level. Additionally, therapeutic endeavours predominantly focus on multi‐functional LDHs and MCTs. Apart from regulating the lactate cascade, these enzymes also exert a crucial influence on other chemical reactions. Consequently, the development of a specific inhibitor targeting histone lactylation necessitates further investigation in our subsequent studies. In addition, the majority of studies substantiate the oncogenic function of histone lactylation in both the initiation and advancement of tumourigenesis. Nevertheless, it is imperative to thoroughly investigate whether histone lactylation may also exert an inhibitory influence on tumour progression, as most modifications exhibit a dualistic impact on carcinogenesis.

In light of the significant role of lactate signalling in diverse cellular processes, it is crucial to conduct thorough investigations into the underlying mechanisms of oncometabolic‐mediated epigenetic reprogramming in future research endeavours. Considering that histone lactylation is indicative of an open chromatin state, which is closely associated with super‐enhancer and 3D‐chromosome architecture, it is warranted to pursue further endeavours aimed at developing a more precise model for epigenetic modulation. By investigating the correlation between histone lactylation and cancer characteristics, we propose that histone lactylation plays a crucial role in predisposing cells to a malignant state, highlighting the significance of identifying innovative therapeutic approaches or dual‐targeting methods to combat lactylation for effective cancer management.

## AUTHOR CONTRIBUTIONS

Meili Li and Peirong Lu planned the project. Yu Zhang and Hang Song collected relevant information and clinical samples. All authors analysed the data. Yu Zhang wrote the paper. All authors discussed the results and commented on the manuscript.

## CONFLICT OF INTEREST STATEMENT

The authors declare no competing financial interests.

## ETHICS STATEMENT

Not applicable.

## Data Availability

All relevant data are available from the authors upon request.
